# Ataxia-televangelist mutated (ATM)/ ATR serine/threonine kinase (ATR)-mediated RAD51 recombinase (RAD51) promotes osteogenic differentiation and inhibits osteoclastogenesis in osteoporosis

**DOI:** 10.1080/21655979.2022.2026729

**Published:** 2022-02-17

**Authors:** Minli Qiu, Liudan Tu, Minjing Zhao, Mingcan Yang, Jun Qi, Ya Xie, Jieruo Gu

**Affiliations:** Department of Rheumatology, The Third Affiliated Hospital of Sun Yat-Sen University, Guangzhou, China

**Keywords:** Osteoporosis, osteoblastogenesis, osteoclastogenesis, RAD51, ATM/ATR

## Abstract

Osteoporosis is a metabolic bone disease that significantly affects the quality of life and can even lead to death. In this study, we aimed to investigate the role of RAD51 recombinase (RAD51) in osteoblast and osteoclast differentiation. We analyzed differentially expressed genes using microarray analysis. The osteogenic differentiation capability was analyzed by alkaline phosphatase (ALP) staining and alizarin red staining assays. Osteogenesis and osteoclast related genes expression was detected using quantitative real-time PCR (qPCR) and Western blotting. The phosphorylation of Ataxia-telangiectasia mutated (ATM) and ATR serine/threonine kinase (ATR) was tested using Western blotting. The effect of RAD51 on osteoporosis was also explored in vivo. The results showed that RAD51 was downregulated in osteoporosis, but upregulated in differentiated osteoblasts. Overexpression of RAD51 enhanced the differentiation of osteoblasts and suppressed the formation of osteoclasts. Furthermore, p-ATM and p-ATR levels were upregulated in osteoblasts and downregulated in osteoclasts. RAD51 expression was reduced by the ATM/ATR pathway inhibitor AZ20. AZ20 treatment inhibited osteoblastogenesis and promoted osteoclastogenesis, whereas RAD51 reversed the effects induced by AZ20. Moreover, RAD51 improved bone microarchitecture in vivo. Taken together, ATM/ATR signaling-mediated RAD51 promoted osteogenic differentiation and suppressed osteoclastogenesis. These findings reveal a critical role for RAD51 in osteoporosis.

## 1. Introduction

Osteoporosis is a metabolic bone disease characterized by osteopenia, deterioration of microarchitecture, and fragility fractures [1]. It is more common in elderly and postmenopausal women [2,3]. Osteoporosis causes pain and spinal deformities and increases the risk of fragility fractures [4]. The etiologies of osteoporosis are complex and include family history, environment, drugs, hormones, and lack of physical activity [5]. Due to the lack of early screening, most osteoporosis cases are undiagnosed and untreated, leading to severe osteoporosis and death [6]. At present, calcium plus vitamin D, estrogen, calcitonin, and etidronate are used for osteoporosis treatment [7]. However, these agents do not cure osteoporosis. Therefore, new therapeutic strategies for osteoporosis require further investigation.

An imbalance in bone homeostasis can cause numerous diseases including osteoporosis. Bone remodeling is associated with the interaction between osteoblast differentiation and osteoclast resorption [8]. Osteoblasts and osteoclasts are highly specialized. They replace termly to maintain a balance between bone formation and degradation [9]. As our understanding of the pathogenesis of osteoporosis increased, we have found that osteoporosis is closely related to impaired osteoblast proliferation and differentiation, as well as the reduction of osteoclasts [10,11]. Therefore, studies on osteoblasts and osteoclasts contribute to the understanding of the mechanisms of osteoporosis and the design of novel therapies.

RAD51 recombinase (RAD51) is an ATPase. Along with the other members of its gene family, RAD51 is also a crucial regulator of DNA fidelity [12]. RAD51 repairs DNA damage accurately and in a timely manner and exerts numerous functions in homologous recombination (HR), DNA replication stress, and meiosis [12,13]. In addition to its effects on HR, RAD51 can support cell survival, regulate break-induced replication, and protect damaged or stalled replication forks [14]. RAD51 is a downstream effector of Ataxia-telangiectasia mutated (ATM)/ ATR serine/threonine kinase (ATR). ATM and ATR are conserved serine/threonine protein kinases that mediate DNA damage responses [15]. Phosphorylation of ATM and ATR initiates RAD51-mediated HR [16]. During meiosis process, ATM suppresses inter-sister recombination mediated by RAD51 [17]. It has been reported that RAD51 is involved in human diseases, such as tumors and Fanconi anemia (FA) [18,19]. Osteoporosis is present in more than half of patients with FA [20]. However, the role of RAD51 and its interaction with ATM/ATR in osteoporosis remains unclear.

In the current study, we aimed to investigated the effects of RAD51 on osteogenic differentiation and osteoclastogenesis. We hypothesized that RAD51 was regulated by ATM/ATR to promote osteogenesis and inhibited osteoclastogenesis. This study provides a basis for the future prospect of RAD51 treating osteoporosis.

## 2. Materials and methods

### 2.1 Microarray analysis

Related microarray GSE100609 was downloaded from the Gene Expression Omnibus (GEO) database (http://www.ncbi.nlm.nih.gov/geo/). The invariant set normalization method was used to normalize spots. The Student’s t-test was used for comparison. Data with p< 0.05 were selected for analysis.

### 2.2 Serum sample collection

The study was approved by the Ethics Committee of the Third Affiliated Hospital of Sun Yat-sen University. Written informed consent was obtained from all participants. In the Third Affiliated Hospital of Sun Yat-sen University, we defined osteoporosis as T-score ≤−2.5, according to the WHO criteria. Healthy controls were defined as those with a T-score ≥−1.0. Nobody had received any treatment for osteoporosis. Patients with malignancies, organ dysfunction, or inflammatory diseases were excluded. Serum samples were collected from 30 patients with osteoporosis and 30 healthy individuals.

### 2.3 Cell culture, osteogenic/ osteoclastic differentiation induction

Murine pre-osteoblasts (MC3T3-E1) and monocytic (RAW264.7) cells were purchased from the Chinese Academy of Medical Sciences (Shanghai, China). MC3T3-E1 cells were maintained in α-MEM (Hyclone, South Logan, UT, USA) supplemented with 10% fetal bovine serum (FBS; Hyclone), 100 μg/mL streptomycin, and 100 μg/mL penicillin (Sigma-Aldrich). RAW264.7 cells were maintained in DMEM (Hyclone) supplemented with 10% FBS, 100 μg/mL streptomycin, and 100 μg/mL penicillin. The cells were cultured at 37°C with 5% CO_2_.

For the induction of osteogenesis, MC3T3-E1 cells were treated with 10 nM dexamethasone (Sigma-Aldrich, St. Louis, MO, USA), 10 mM β-glycerophosphate (Sigma-Aldrich), and 50 mM ascorbic acid (Sigma-Aldrich) for 2 weeks.

To induce osteoclastogenesis, RAW 264.7 cells were treated with 100 ng/mL M-CSF (R&D, Minneapolis, MN) for 2 d alone and then together with 50 ng/mL Receptor Activator for Nuclear Factor-κ B Ligand (RANKL; R&D, Minneapolis, MN) for 3 d.

To inhibit the effects of the ATM/ATR pathway, the cells were treated with 5 nM AZ20 (Beyotime, Shanghai, China).

### 2.4 Cell transfection

RAD51 sequences were cloned into pcDNA3.1 to construct RAD51 overexpressing plasmids. The pcDNA3.1 vector was used as the negative control. The cells were seeded into 6-well plates and transfected with pcDNA3.1 and RAD51 plasmids using Lipofectamine 3000 (Invitrogen, Carlsbad, CA, USA). After transfection for 48 h, the expression of RAD51 was detected using quantitative real-time PCR (qPCR).

### 2.5 Alkaline phosphatase (ALP) staining assay

ALP staining assay was performed as described previously [21]. After the induction of osteogenesis, MC3T3-E1 cells were seeded into 24-well plates and fixed with 4% paraformaldehyde for 15 min. The cells were incubated with 200 µL of propanol for 15 min, 200 µL of ALP incubation solution for 6 h, 200 µL of cobalt nitrate for 15 min, and 200 µL of ammonium sulfide for 5 min. Stained cells were visualized under a microscope (Olympus, Tokyo, Japan). The optical density value at 490 nm was measured using a microplate reader (Thermo Fisher Scientific, Waltham, MA, USA).

### 2.6 Alizarin red staining (ARS) assay

ARS assay was conducted as described previously [21]. MC3T3-E1 cells were digested, seeded into 24-well plates and fixed with 4% paraformaldehyde for 15 min. Then, the MC3T3-E1 cells were stained with 200 µL of ARS solution for 15 min. Stained cells were photographed under a microscope. Absorbance at 570 nm was measured using a microplate reader.

### 2.7 qPCR

Total RNA was extracted using the TRIzol reagent (Invitrogen). The integrity of RNA was determined by agarose gel electrophoresis, and RNA concentration was measured by ultraviolet spectrophotometer. Approximately 1 µg of RNA was used for reverse transcription using the RevertAid First Strand cDNA Synthesis Kit (Thermo Fisher Scientific, Inc.). qPCR was conducted using Power qPCR PreMix (SYBR Green) (GeneRay, Shanghai, China) on a LightCycler qPCR reaction system (Roche, Basel, Switzerland) under the following conditions: 95°C for 10 min, 45 cycles of 95°C for 10s, 60°C for 20s, and 72°C for 15s. GAPDH served as an internal control. The relative mRNA expression (fold change) was calculated using the 2^−ΔΔCt^ method. Primers sequences were listed in Table 1.

**Table 1.** Primers sequences used in qPCR

Name	Forward sequence (5’-3’)	Reverse sequence (5’-3’)
RAD51	CGCCTTGGCTTGTCCAGAT	GGTGCTAGTGGCATTTGGATG
ALP	ACCTGAGTGCCAGAGTGA	CTTCCTCCTTGTTGGGTT
RUNX2	TCTTAGAACAAATTCTGCCCTTT	TGCTTTGGTCTTGAAATCACA
BMP4	CTCCCAAGAATCATGGACTG	AAAGCAGAGCTCTCACTGGT
TRAP	ACCATGACCACCTTGGCAATGTCTC	ATAGTGGAAGCGCAGATAGCCGTT
NFATC1	CTCGAAAGACAGCACTGGAGCAT	CGGCTGCCTTCCGTCTCATAG
Cathepsin K	TATGACCACTGCCTTCCAATAC	GCCGTGGCGTTATACATACA
Ltgb3	CCGGGGGACTTAATGAGACCACTT	ACGCCCCAAATCCCACCCATACA
GAPDH	GAAGGTGAAGGTCGGAGTC	GAGATGGTGATGGGATTTC

### 2.8 Western blot

Total RNA was extracted using RIPA lysis buffer (Beyotime) for 1 h on ice. The protein concentration was detected using a BCA trace protein detection kit (Jiancheng, Nanjing, China). Next, each protein sample (30 µg) was separated using 10% SDS-PAGE and transferred onto PVDF membranes (Millipore, Billerica, MA, USA). Membrane blocking was performed using WB blocking solution (Jiancheng). Subsequently, the membranes were incubated with primary antibodies specific for ALP, RUNX2, BMP4, TRAP, NFATC1, cathepsin K, ltgb3, p-ATM, p-ATR, and GAPDH (Abcam, Cambridge, MA, USA) at 4°C overnight. After washing using TBST, membranes were incubated with secondary antibodies at 25°C for 2 h. The protein was developed using a low-background luminescence enhanced chemiluminescence (ECL) detection kit (Jiancheng). GAPDH was used for normalization.

### 2.9 In vivo assays

Male Sprague Dawley mice (n = 36, 8 weeks old, 220 ± 20 g) were obtained from Nanjing Medical University (Nanjing, China). Mice were randomly assigned to the negative control (NC), hindlimb unloading (HU), HU+pcDNA3.1, or HU+RAD51 groups. Mice in the HU group were suspended by the tail for 4 weeks. Mice in the HU group were intraperitoneally injected with PBS, pcDNA3.1, and RAD51 (n = 10 for each group). Moreover, mice were suspended by the tail with forefeet touching the ground and provided with free access to food. Mice in the NC group were injected with PBS. After 4 weeks, all mice were euthanized. The bone microarchitecture was determined using a micro-CT scanner (PerkinElmer, MA, USA).

### 2.10 Statistical analysis

GraphPad Prism 7.0 (GraphPad Software, CA, USA) was utilized to analyze all data in this study. Data are presented as means ± standard deviation (SD). Unpaired Student’s t-test was used to compare data between two groups, while one-way analysis of variance (ANOVA) coupled with Tukey’s post hoc test was used to compare data among multiple groups. Statistical significance was set at P-value less than 0.05.

## 3. Results

We investigated the role of RAD51 in osteoporosis and the molecular mechanism. We analyzed osteogenesis by ALP staining assay, ARS assay and detecting osteogenic related factors. Osteoclastogenesis was evaluated by detecting osteoclast related factors. We hypothesized that RAD51 was regulated by ATM/ATR to promote osteogenesis and inhibited osteoclastogenesis. This study provides a basis for RAD51 to become the target of treatment osteoporosis.

### 3.1 RAD51 expression was downregulated in patients with osteoporosis

**Figure 1.**
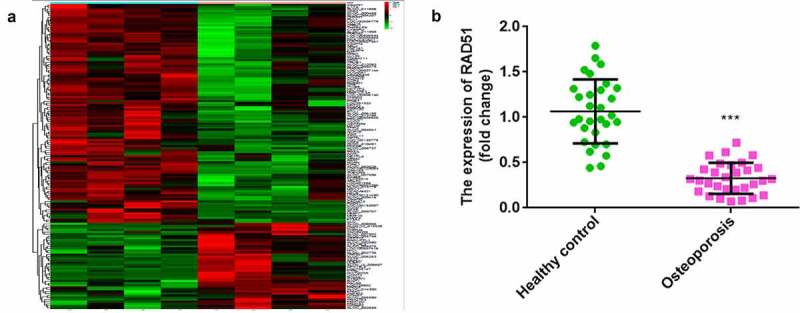
Downregulated RAD51 in osteoporosis. (a) Microarray analysis was performed to investigate the abnormal expression of mRNAs between the control and osteoporosis groups. Red indicates high levels, and green indicates low levels. (b) The expression levels of RAD51 in the serum of patients with osteoporosis and healthy individuals were determined by qPCR. ***P < 0.001.

As shown in Figure 1a, bioinformatics analysis indicated that 150 mRNAs were dysregulated between the control and osteoporosis groups. A total of 107 mRNAs were upregulated and 43 mRNAs were downregulated in osteoporosis. RAD51 was one of the mRNAs downregulated in osteoporosis. Furthermore, qPCR illustrated that RAD51 expression was significantly decreased in the serum of patients with osteoporosis (Figure 1b).

### 3.2 Overexpression of RAD51 promoted osteogenic differentiation

**Figure 2.**
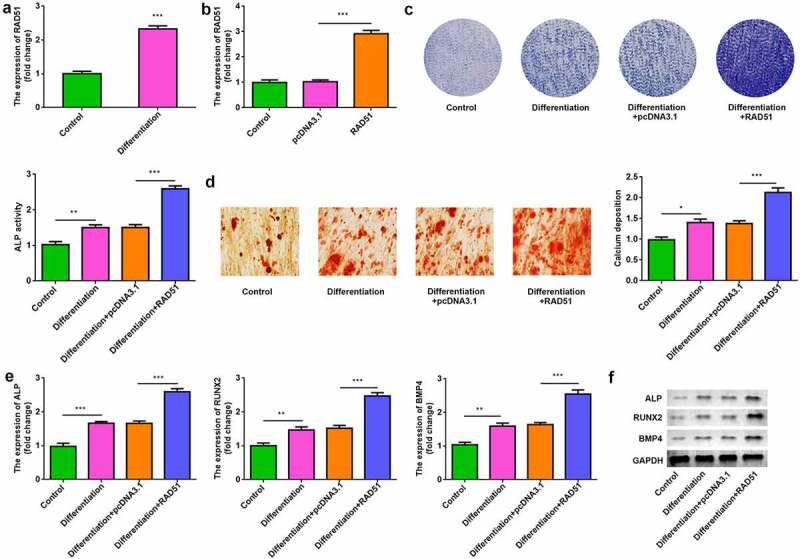
RAD51 promoted osteogenic differentiation. (a) The expression levels of RAD51 in undifferentiated and differentiated cells were determined by qPCR. (b) Transfection efficiency was assessed by qPCR. (c) The effects of RAD51 on ALP activity were assessed by ALP staining. (d) Calcium deposition was assessed by an ARS assay. (e) The expression levels of ALP, RUNX2, and BMP4 were determined using qPCR. (f) The expression levels of ALP, RUNX2, and BMP4 were determined using Western blot. ***P < 0.001. **P < 0.01. *P < 0.05.

To assess the role of RAD51 in osteoblastogenesis, osteogenic differentiation of MC3T3-E1 cells was induced. RAD51 expression was up-regulated in the differentiation group (Figure 2a). After transfection, RAD51 levels were significantly increased in the RAD51 group compared with the pcDNA3.1 group (Figure 2b). ALP activity and calcium deposition were significantly higher in differentiated cells, which were significantly increased by RAD51 overexpression (Figure 2c and d). The results of qPCR and Western blotting indicated that the expression levels of ALP, RUNX2, and BMP4 were markedly higher in differentiated osteoblasts and were further significantly increased by RAD51 overexpression (Figure 2e and f).

### 3.3 Overexpression of RAD51 suppressed osteoclastogenesis

**Figure 3.**
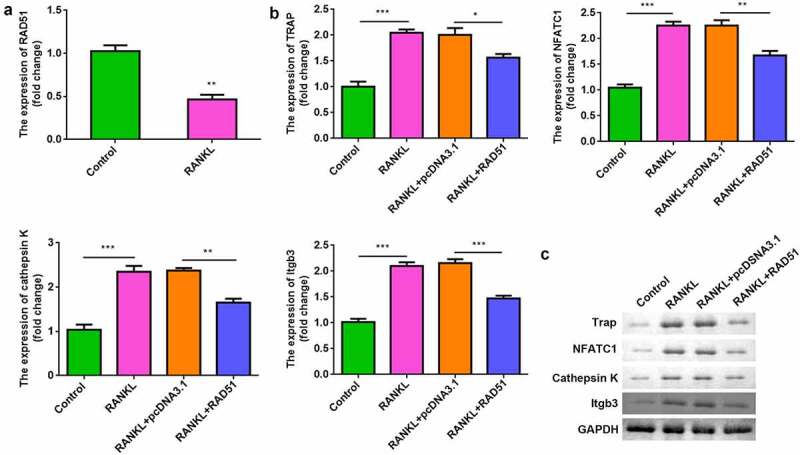
RAD51 suppressed osteoclastogenesis. (a) The expression level of RAD51 was determined in RAW264.7 cells treated with or without RANKL by qPCR. (b) The mRNA expression levels of TRAP, NFATC1, cathepsin K, and Itgb3 were determined using qPCR. (c) The protein expression levels of TRAP, NFATC1, and cathepsin K were determined using Western blot. ***P < 0.001. **P < 0.01. *P < 0.05.

While assessing the effects of RAD51 on osteoclastogenesis, we found that RANKL treatment significantly downregulated RAD51 in RAW264.7 cells (Figure 3a). Moreover, overexpression of RAD51 alleviated the RANKL-induced increase in the mRNA and protein expression of TRAP, NFATC1, cathepsin K, and Itgb3 (Figure 3b and c).

### 3.4 RAD51 expression was regulated by ATM/ATR signaling

**Figure 4.**
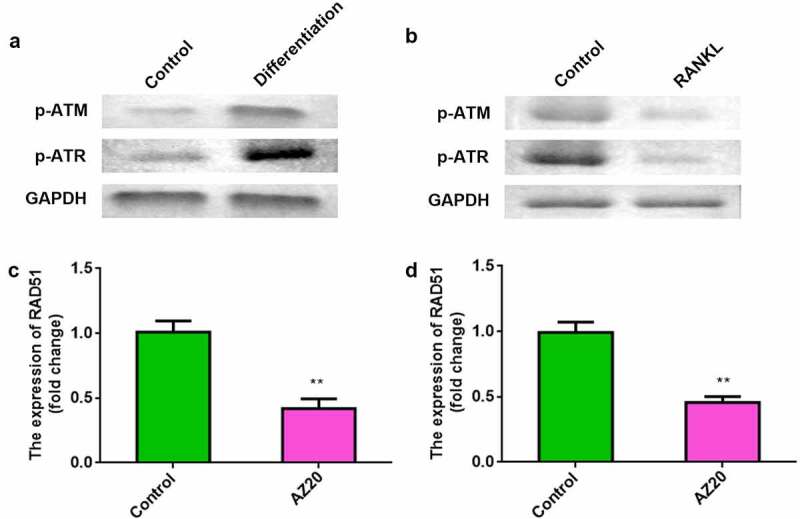
RAD51 level was positively regulated by ATM/ATR signaling. (a) The protein expression levels of p-ATM and p-ATR in the osteoblasts were determined using Western blot. (b) The protein expression levels of p-ATM and p-ATR in the osteoclasts were determined using Western blot. (c) Expression levels of RAD51 in osteoblasts treated with AZ20. (d) Expression levels of RAD51 in osteoclasts treated with AZ20. **P < 0.01.

Next, we determined the level of ATM/ATR signaling in osteoblasts and osteoclasts. The levels of p-ATM and p-ATR were elevated in osteoblasts (Figure 4a). Conversely, p-ATM and p-ATR levels were reduced in osteoclasts (Figure 4b). To explore the effects of ATM/ATR on RAD51, AZ20 was used to inhibit the ATM/ATR pathway. Furthermore, AZ20, an inhibitor of the ATM/ATR pathway, resulted in reduced levels of RAD51 in both osteoblasts and osteoclasts (Figure 4c and d).

3.5 RAD51 facilitated osteoblastogenesis and inhibited osteoclastogenesis mediated by the ATM/ATR pathway

**Figure 5.**
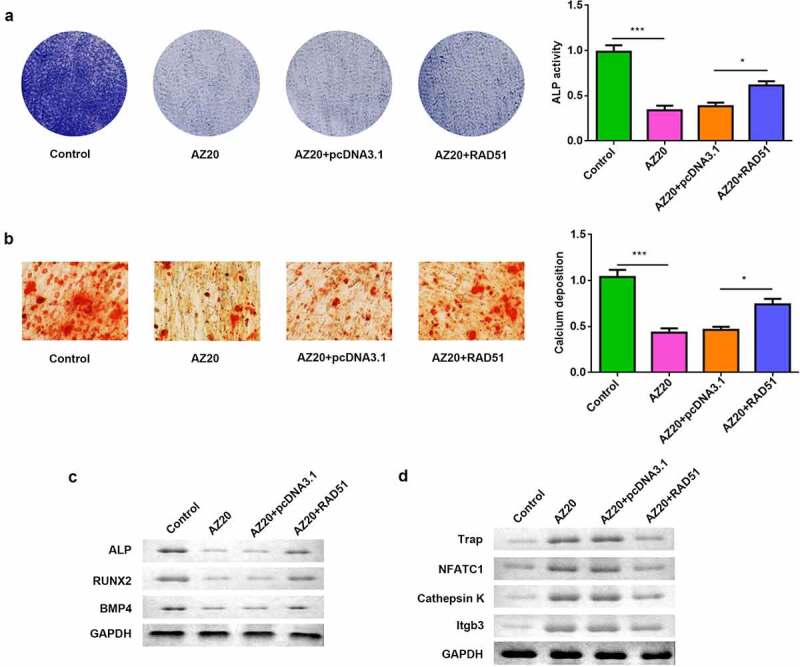
ATM/ATR-RAD51 axis facilitate osteoblastogenesis and suppress osteoclastogenesis. (a) Osteoblastogenesis was assessed by ALP staining assay. (b) Calcium deposition was assessed by an ARS assay. (c) The levels of ALP, RUNX2, and BMP4 were determined by Western blot. (d) The levels of TRAP, NFATC1, and cathepsin K were determined by Western blot. ***P < 0.001. *P < 0.05.

To confirm that the effects of RAD51 were mediated by ATM/ATR, osteogenically differentiated MC3T3-E1 cells were treated with AZ20 and RAD51 overexpression. AZ20 inhibited ALP activity, and overexpression of RAD51 abolished this inhibition (Figure 5a). The results of the ARS assay showed that calcium deposition was reduced by AZ20 and rescued by RAD51 (Figure 5b). The protein levels of ALP, RUNX2, and BMP4 were decreased by AZ20 treatment, which was abrogated by RAD51 overexpression (Figure 5c). On the other hand, osteoclastogenic RAW264.7 cells were also exposed to AZ20 and transfected with RAD51 plasmids. The protein levels of TRAP, NFATC1, and cathepsin K were elevated by AZ20, which was reversed by RAD51 overexpression (Figure 5d).

### 3.6 RAD51 alleviated osteoporosis in vivo

**Figure 6.**
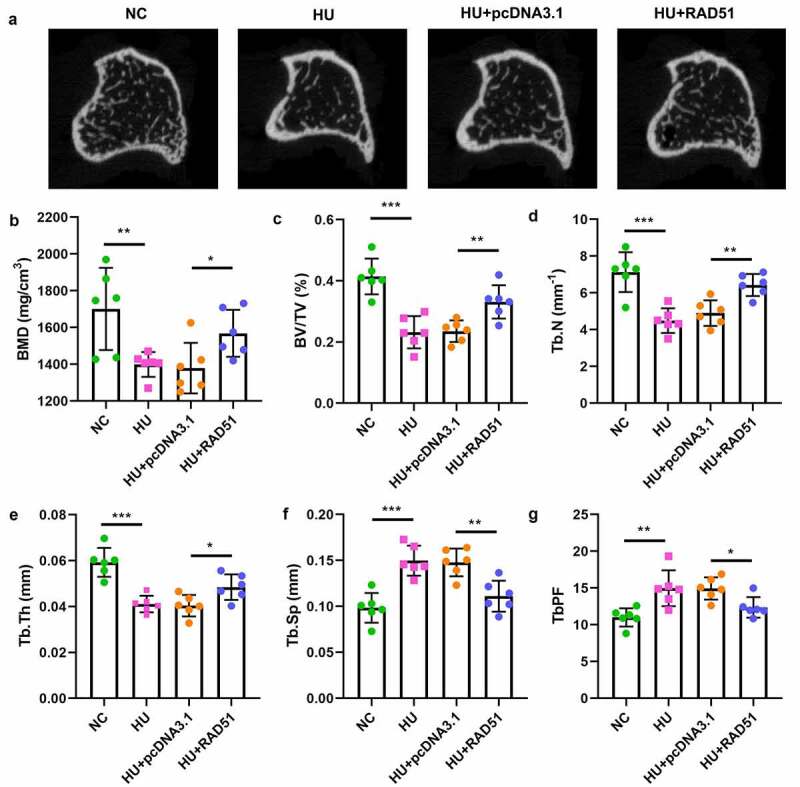
RAD51 suppressed osteoporosis in vivo. (a) The representative micro-CT sectional femoral images. (b-g) The bone microarchitecture parameters, such as BMD, BV/TV, Tb.N, Tb.Th, Tb.Sp, and Tb.PF, were analyzed by micro-CT. ***P < 0.001. **P < 0.001. *P < 0.05.

To further verify the potential role of RAD51 in osteoporosis, we performed an in vivo assay. As shown in Figure 6a and b, the bone mineral density (BMD) in the HU group was lower than that in the NC group; however, RAD51 alleviated the development of osteoporosis. Moreover, RAD51 caused significant increases in the values of bone volume per total volume (BV/TV), trabecular number (Tb.N) and trabecular thickness (Tb.Th), and decreased the values of trabecular separation (Tb.Sp) and trabecular bone pattern factor (Tb.PF) (Figure 6c-g).

## 4. Discussion

In the present study, RAD51 was found to be dysregulated in osteoporosis. Functional experiments showed that RAD51 overexpression promoted osteogenic differentiation and inhibited osteoclast differentiation. The effects of RAD51 were mediated by ATM/ATR signaling.

An imbalance in bone remodeling is a direct cause of bone loss and eventually leads to osteoporosis. Osteoblasts promote the formation of bone and enhance bone mineralization, whereas osteoclasts promote bone resorption and digest bone minerals [22]. Previous studies have reported that promoting osteogenic differentiation and/or inhibiting osteoclast differentiation can alleviate osteoporosis. For example, circular RNA_0062582 promotes bone mineralization and levels of osteogenic genes [23]. Vitamin K2 facilitates osteogenic differentiation and mineralization [24]. In addition, miR-125-5p and LGR4 play essential roles in the regulation of osteoclastogenesis [25,26]. However, little is known about the role of RAD51 in osteoporosis.

It is well known that DNA damage causes gene mutations, chromosome rearrangements, and genomic instability, leading to tumorigenesis and progression, development of inflammatory diseases, and chronic diseases [27–29]. The DNA damage response plays an important role in bone remodeling and is associated with osteoporosis and osteosclerosis [30]. DNA damage in the elderly causes mesenchymal stem cells to differentiate into fat cells rather than into osteoblasts [31]. RAD51 is a DNA damage response protein that plays a key role in HR and helps increase tolerance to DNA damage [32]. Previous studies have focused on the role of RAD51 in tumors [33,34]. Here, we speculated that RAD51 is involved in the development of osteoporosis. In this study, the expression of RAD51 was found to be decreased in patients with osteoporosis. Next, we examined the role of RAD51 in osteoblasts and osteoclasts. Overexpression of RAD51 facilitated osteoblastogenesis and suppressed osteoclastogenesis, suggesting that RAD51 might alleviate osteoporosis. Moreover, RAD51 suppressed osteoporosis in vivo, suggesting that RAD51 is beneficial for suppressing the development of osteoporosis. It is the first study to show that RAD51 is involved in the progression of osteoporosis.

ATM and ATR are DNA damage sensor, which are activated at sites of human DNA damage [15,35]. Once activated, ATM/ATR phosphorylation contributes to maintaining the stability of stalled replication forks, helps to repair DNA damage and coordinate DNA repair with other DNA metabolic events [36,37]. The effects of ATM/ATR are focused on ataxia-telangiectasia-like disorders and malignancies [38,39]. RAD51 is a downstream effector of ATM/ATR. A previous study has reported that the RAD51 function is regulated by ATM/ATR via phosphorylation of PALB2 [40]. However, the involvement of ATM/ATR and the relationship between RAD51 and ATM/ATR in osteoporosis remains unknown. In the current study, we found that p-ATM and p-ATR were highly expressed in osteoblasts, whereas their expression was low in osteoclasts. After the inhibition of ATM/ATR signaling, RAD51 expression was downregulated. The results of functional experiments indicated that inhibition of ATM/ATR suppressed osteogenic differentiation and facilitated osteoclast differentiation, whereas overexpression of RAD51 reversed these effects. These data suggest that the progression of osteoporosis alleviated by RAD51 is regulated by upstream ATM/ATR signaling.

This study also has limitation. This study lacks research and discussion of the clinical significance of this paper. Our future study will explore the clinical effect of RAD51 on osteoporosis.

## 5. Conclusion

In short, the expression of RAD51 is decreased in osteoporosis. We found for the first time that ATM/ATR regulated RAD51 to facilitate osteoblastogenesis and suppress osteoclastogenesis. Moreover, RAD51 alleviated osteoporosis in mice. These findings suggest that RAD51 may be a novel target for osteoporosis therapy.

## Research highlights

1. RAD51 is downregulated in patients with osteoporosis.

2. RAD51 promotes osteoblast differentiation and inhibits osteoclast formation.

3. ATM/ATR signaling regulates RAD51 expression.

4. RAD51 alleviates osteoporosis in hindlimb unloading mice.

5. ATM/ATR-mediated RAD51 improves osteoporosis.
